# Target Classification in Synthetic Aperture Radar Images Using Quantized Wavelet Scattering Networks †

**DOI:** 10.3390/s21154981

**Published:** 2021-07-22

**Authors:** Raghu G. Raj, Maxine R. Fox, Ram M. Narayanan

**Affiliations:** 1 U.S. Naval Research Laboratory, Radar Division, Washington, DC 20375, USA; raghu.raj@nrl.navy.mil (R.G.R.); maxine.fox@nrl.navy.mil (M.R.F.); 2 Department of Electrical Engineering, The Pennsylvania State University, University Park, State College, PA 16802, USA

**Keywords:** adaptive wavelet scattering network, backpropagation, classification, convolutional neural networks, MSTAR, synthetic aperture radar, wavelet scattering network

## Abstract

The need to classify targets and features in high-resolution imagery is of interest in applications such as detection of landmines in ground penetrating radar and tumors in medical ultrasound images. Convolutional neural networks (CNNs) trained using extensive datasets are being investigated recently. However, large CNNs and wavelet scattering networks (WSNs), which share similar properties, have extensive memory requirements and are not readily extendable to other datasets and architectures—and especially in the context of adaptive and online learning. In this paper, we quantitatively study several quantization schemes on WSNs designed for target classification using X-band synthetic aperture radar (SAR) data and investigate their robustness to low signal-to-noise ratio (SNR) levels. A detailed study was conducted on the tradeoffs involved between the various quantization schemes and the means of maximizing classification performance for each case. Thus, the WSN-based quantization studies performed in this investigation provide a good benchmark and important guidance for the design of quantized neural networks architectures for target classification.

## 1. Introduction

Feature extraction and classification are essential ingredients in imagery analysis in myriad applications: remote sensing, military, nondestructive testing, ultrasound, medical, cell analysis, etc. In particular, image classification is the process of organizing images into different classes based on the output of feature extraction operators applied to images. There are innumerable approaches to feature extraction, a necessary precursor to classification, including decision-theoretic approaches using quantitative descriptors such as length, area, and texture [1,2]; structural approaches using qualitative descriptors, such as relational descriptors [3]; projection of data into fixed basis sets, such as wavelets [4] and Zernike polynomial moments [5], or adaptive basis sets [6]. Other examples include robust edges and corners that are popular in computer vision, blind synthesis of template classes by using singular value decomposition, Karhunen–Loeve Transform [7,8] and estimation theoretic templates [9], motion-based covariance matrix-based features for multi-sensor architectures [10], and finally micro-Doppler- [11] and vibrometry-based [12] features that have applications in radar-based sensing systems. The advent of deep neural networks, a variant of which is the focus of our work, has systematized to a large extent the process of feature extraction and classification.

Prior to feature extraction, several pre-processing steps are required to recognize targets/objects embedded within an image. For example, a common first step for image analysis is image segmentation [13]. Depending on the available independent knowledge about an image, specialized approaches to segmentation to isolate regions containing features of interest may be applied [14]. Another technique, image clustering, can be used to divide images, subregions, or even features into similar groups or “clusters”. For multiband images and multi-dimensional data, cluster analysis is usually encountered to find the clusters and fit boundaries between them that can be used for classification.

Neural networks, which are used to analyze and process images, have been shown to largely circumvent excessive dependence on pre-processing issues such as segmentation, etc., as described above. In particular, neural networks can be based on task or abstraction levels. Tasks include spatial or spectral filtering, feature extraction, object detection and recognition, and image understanding. Abstraction levels include pixels, features, measurement values, object relationships, and scene types.

Overfitting is a common problem in image classification because there are usually too few training samples, resulting in a model with poor generalization performance. One solution to overfitting is to use data augmentation, which is a technique to generate more training data from the current training set. It is an artificial way to boost the size of the training set, reducing overfitting. Data augmentation is typically done by data transformations and processing, such as rotation, shifting, resizing, adding noise, contrast change, etc. Data augmentation is only performed on the training data, not on the validation or test set.

Recently, convolutional neural networks (CNNs) have achieved state-of-the-art performance on several benchmark tasks in the computer vision literature. This suggests the possibility of applying CNNs to high resolution images for detecting and classifying features and objects. CNNs are a supervised classification model with many more free model parameters compared to other supervised methods. As a result, CNNs need large quantities of training data [15]. This is a major challenge for many applications, wherein the collection of training data is time consuming and expensive.

CNNs have found application to synthetic aperture radar (SAR) in recent works using datasets such as the publicly available MSTAR dataset [16,17,18,19,20,21]. These studies typically used predefined network architectures that can contain many learnable parameters; other available architectures can contain upwards of a million parameters [22,23,24], which require significant amounts of memory. To reduce needed memory, the quantization of generalized or otherwise large CNNs has been studied for the classification of optical datasets [25,26]. Despite promising results, extensions to other datasets and network architectures are not proven easily due to their variability. The success of a CNN design is dependent upon many factors, including the arrangement of layers the number and initialization of learnable parameters in each layer, the learning rate and update methods implemented during training, and the size and complexity of the available training data. Given such variability, the development of a network for benchmarking these designs would prove useful.

Several authors have implemented wavelet scattering networks (WSNs) to demonstrate the paramount properties of CNNs, particularly convolution, nonlinearity, and the layered architecture [27,28,29]. With far fewer design parameters and lower memory requirements, the similar functionality of a WSN may be utilized as a benchmark for comparing quantization schemes among different CNN architectures in future work.

The foundation of a WSN, the wavelet scattering transform, is itself an effective instrument in feature extraction due to its provision of translation invariance, stability, and the ability to linearize small diffeomorphisms that result from its layered architecture of scattering wavelets. It is even used as a preprocessing measure wherein a WSN performs preliminary feature extraction prior to the training of a deep neural network (DNN) for localization [30]. The freedom of choosing an appropriate kernel of a linear transform has been exploited fully, which is generally known as the adaptive wavelet transform [31]. Several levels of adaptivity were investigated in detail. To overcome the problem that the noise cannot generally be predicted in a noisy signal, an adaptive filter based on the wavelet transform method was implemented [32]. The results show that the Hopefield neural network adaptive filter model based on wavelet transform achieved the best denoising effect. The adaptive wavelet invariant moments (AWIM) formulation was proposed and developed to ensure that the discrete wavelet transform (DWT) coefficients were both translation and scale invariant [33]. This technique was successfully implemented for scale-invariant texture identification.

The WSN in [27] computed a translation-invariant image representation which was stable to deformations and preserved high-frequency information for classification. It was implemented by cascading wavelet transform convolutions with nonlinear modulus and averaging operators. A detailed mathematical analysis of WSNs explained important properties of deep convolution networks for classification. A windowed scattering transform was defined by a local integration, and, as the window size increased, it converged to a wavelet scattering transform that was translation invariant [28].

An architecture employing a deep WSN to extract translation- and rotation-invariant features was used by a conditional random field to perform scene segmentation on aerial images recorded from an unmanned aerial vehicle [34]. A parametric log transformation with dual-tree complex wavelets was proposed and implemented to extract translation-invariant representations from a multi-resolution image [35]. The parametric transformation improved the pruning algorithm, while the dual-tree wavelets improved the computational efficiency of the network. 

A two-layer WSN was presented for object classification [29]. This scattering transform computed a spatial wavelet transform on the first layer and a new joint wavelet transform along spatial, angular, and scale variables in the second layer. By applying a WSN in various color spaces, color texture classification was achieved, with the opponent RGB-based WSN outperforming other color spaces [36].

A framework was implemented to classify cell images based on WSNs and random forests [37]. The WSN computed rotation-invariant wavelet coefficients as representations of cells images, while a random forest classifier was trained to predict the pattern label of a cell image from six classes. The feasibility of deep WSN-based features for classification of ultrasound images acquired in a muscle computer interface was investigated [38]. Deep learning-based feature extractors were seen to be invariant to translation, rotation, and slight deformations, thereby preserving motion classification accuracy.

A wavelet scattering transform was used to extract reliable features that are stable to small deformation and are rotation-invariant when applying an artificial neural network (ANN) to indoor fingerprinting-based localization, where the signal is inherently unstable [30]. The extracted features were used by a DNN model to predict the location. An automatic target recognition method for SAR images was developed based on a super-resolution generative adversarial network (SRGAN) and deep convolutional neural network (DCNN) [39]. The approach was able to suppress background clutter, enhance target feature characterization ability, and achieve automatic target classification and recognition.

The roto-translation properties of the WSN were incorporated in a convolutional architecture to construct a rotation invariant CNN for image classification [40]. Another scale- and rotation-invariant feature extraction method, the speeded-up robust features (SURF) is a local feature detector and descriptor that utilizes multi-scale representation based on box filters [41]. The binary robust independent elementary features (BRIEF) descriptor improves upon SURF by reducing computation [42].

Complex-valued CNNs have been proposed which yield improved performance over their real-valued counterparts, especially in those with bigger kernel sizes [43]. These can be likened to nonlinear multiwavelet packets, thus making the mathematical analysis from the signal processing perspective available for a rigorous formulation of the properties of complex-valued convolutional networks. Moreover, these are more relevant because many images, especially SAR images, are in complex form.

Expanding upon our preliminary work in [44], this paper systematically explores the application of quantized WSNs to target classification of SAR imagery for a large range of SNR conditions; in particular, we used the MSTAR SAR dataset to validate the techniques presented in this paper. After presenting a quantitative description of the implementation of the important quantization schemes, a detailed study was conducted on the tradeoffs involved between the various quantization schemes and the means of maximizing classification performance for each case. Finally, due to its structural similarity with CNNs, the WSN-based quantization studies performed in this paper may provide a good benchmark and important guidance for the design of quantized CNN-based neural networks.

The remainder of this paper is organized as follows. Section 2 presents a brief introduction of the basics of WSNs and the windowed scattering transform. Section 3 explores the unique architecture of the WSN and the development of the quantization method and scales. Section 4 describes the methodology for the application of WSNs for classification of the MSTAR dataset. Section 5 presents the results and discussion thereof. Conclusions and recommendations for future work on this topic are presented in Section 6.

## 2. Wavelet Scattering Networks Fundamentals

The WSN shares the key properties of a CNN, primarily convolution, nonlinearity, and layer-wise architecture. A WSN is a windowed scattering transform that extracts features at multiple resolutions using scattering wavelets, a subset of wavelet filters that aid in achieving translation invariance, stability, and the linearization of small diffeomorphisms. Daughter wavelets are constructed from a mother wavelet, ψ, at various scales and orientations. For a scaling factor of $2^{j}$ and rotation angle of θ, a 2D daughter wavelet is

(1) $$\psi_{j.\theta }=2^{2j}\psi \left(2^{j}r_{\theta }^{-1}\vec{u}\right),$$

where $r_{\theta }$ is the rotation matrix and the position vector $\vec{u}={\left[x,y\right]}^{T}$. For convenience, this paper uses the notation $\psi_{\lambda }$, where λ≡j,θ indicates a combination of scale and orientation for the remainder of this section, which provides relevant background on the scattering transform, reproduced in part from [26] for better understanding of the WSN architecture during the discussion of the quantization scheme below.

**Figure 1:**
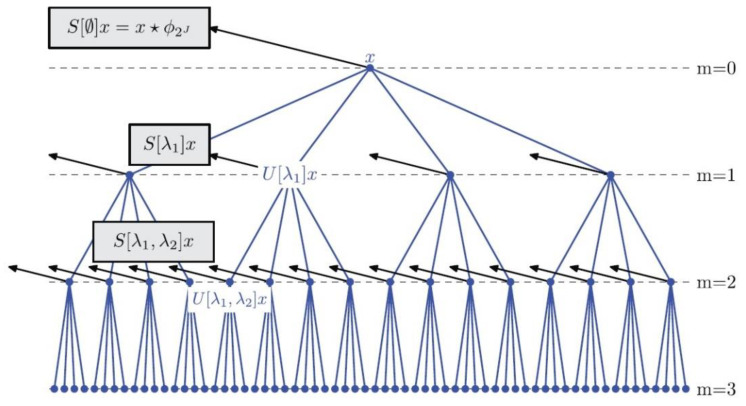
Structure of a wavelet scattering network which computes the windowed scattering transform. Reprinted with permission from ref. [27]. Copyright © 2013 IEEE.

A WSN is a windowed scattering transform, consisting of multiple windowed scattering propagators, typically of multiple scattering orders, like that shown in Figure 1 for a maximal scattering order of M=3. The scattering propagators are formed using a set of daughter wavelets constructed via (1), and the windowing is performed by a son wavelet.

The collection of daughter wavelets, $\psi_{\lambda }$ for all λ∈Λ, defined for J scales and L orientations at Q wavelets per octave, act as high-pass filters. The scale of each is $2^{j/Q}$ for unique j=0,1,…,J−1, where Q acts as a quality factor for the employed filter bank; the quality factor is set to unity in this work, as in the example in the ScatNet documentation [45]. In addition, the orientations, $\theta \in \Theta ={\left\{l\pi /L\right\}}_{l=0}^{L-1}$.

The Littlewood–Paley wavelet transform [46,47] of an input image, X, with a daughter wavelet, $\psi_{\lambda }$ (wherein each element of X is convolved) is subsampled according to its frequency bandwidth

(2) $$W\left[\lambda \right]X={↓}_{2^{d}}\left(\psi_{\lambda }*X\right)$$

where ${↓}_{2^{d}}\left(Z\right)$ denotes the subsampling operation on Z. The log_2_ subsampling rate d is determined as a function of the log_2_ filter resolution, $r_{\psi_{\lambda }}$, and the resolution of the input at the *m*th scattering order, $r_{X}^{\left(m\right)}$, such that

(3) $$d=\max\left\{0,r_{\psi_{\lambda }}-r_{X}^{\left(m\right)}-\zeta \right\}$$

where $r_{\psi_{\lambda }}=j/Q$ and ζ is the oversampling factor. The log_2_ resolution of the output is

(4) $$r_{X}^{\left(m+1\right)}=r_{X}^{\left(m\right)}+d$$

Equation (2) is not translation invariant; therefore, similar to the non-linear activation functions in the CNN, it is passed through a non-linear function for demodulation. The modulus of the output of Equation (2) may be used for this purpose. The complete process of the wavelet transform and its demodulation is described by the scattering operator U[λ], given by

(5) U[λ]X=|W[λ]X|.

A scattering propagator applies Equation (5) at each λ along a path $p=\left(\lambda_{1},\lambda_{2},\ldots \right)$ where each $\lambda_{f}$ in p is unique. For an *m*th-order path, i.e., a path of length m, the scattering propagator is defined as

(6) $$U\left[p\right]X=U\left[\lambda_{m}\right]\ldots U\left[\lambda_{2}\right]U\left[\lambda_{1}\right]X,$$

where U[Ø]=I and I is the identity matrix. In a WSN, the paths in the set of paths, P, that define a scattering propagator are unique and vary in length between 0 and the maximal scattering order, M. The number of paths in P is restricted so that only filters of increasing scale can be used, i.e., $j_{m}=j_{m-1}+Q$.

To form a windowed scattering propagator, a son wavelet, $\phi_{J}$, is constructed from a father wavelet, ϕ, such that

(7) $$\phi_{J}=2^{-2(J-1)}\phi \left(2^{-(J-1)}\vec{u}\right),$$

which acts as a low-pass filter. (Note that, if Q≠1, then the scaling of $\phi_{J}$ is $2^{\left(J-1\right)/Q}$.) The windowed scattering propagator subsamples the convolution of Equation (7) with the scattering propagator in Equation (6), i.e.,

(8) $$S_{J}\left[p\right]X={↓}_{2^{d}}\left(U\left[p\right]X*\phi_{J}\right).$$

Note that the path length of the windowed scattering propagator is the same as the scattering propagator.

**Figure 2:**
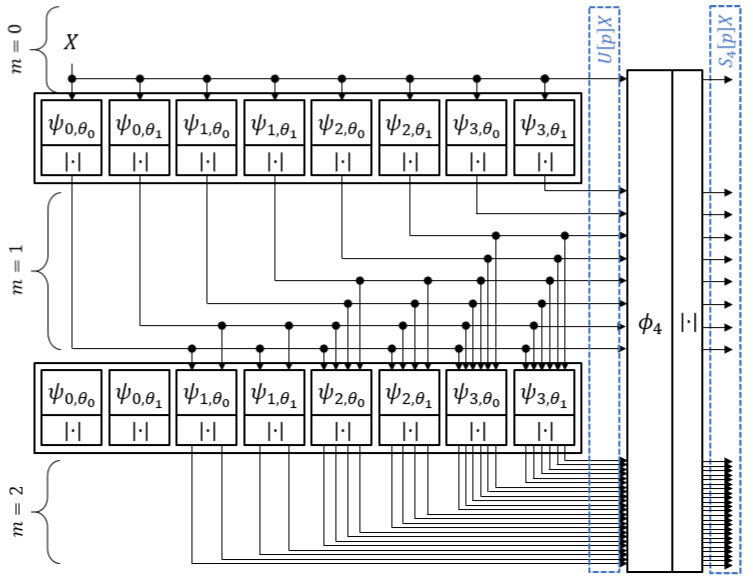
WSN paths with J=4, *L* = 2, and *M* = 2.

Figure 2 provides an example of the paths found in P for J=4, L=2, Q=1, and M=2. Note that these paths overlap: For example, the path $p=\left(\left(0,\theta_{1}\right)\right)$ is part of the path $p=\left(\left(0,\theta_{1}\right),\left(1,\theta_{2}\right)\right)$. The output from each U[p] is passed to $\phi_{4}$, as well as to all orientations of the high-pass filters with scales $2^{j}$, such that $j_{m-1}+1\leq j<J$, where $2^{j_{m-1}}$ is the scale of the most recent filter along the path.

Each $S_{J}\left[p\right]X$ is a feature map akin to those output from the convolutional layers of a CNN and may be input to a classifier, such as a support vector machine (SVM) or the stable SoftMax function, following additional processing.

The combinations of M, J, and L implemented in this section are provided in Table 1. Three and five scales were used to understand the effect of both the scale, as well as the network complexity. Both scales were used for M=0 networks to better understand the effect of the scale in $\phi_{J}$ on the output, as they should behave as CONV layers with one filter. For *M* > 0, differing J and L were implemented to explore the effect of network complexity, particularly with the application of the updates during backpropagation. Note that, in the ScatNet framework, at M=1, all daughter wavelets $\psi_{\lambda }$ are utilized once at *r* = 0 and the windowing function $\phi_{J}$ at multiple resolutions depending upon the value of J; at M=2, all $\psi_{\lambda }$ with *j* > 2 are utilized at more than one resolution.

## 3. Quantization of a Wavelet Scattering Network

The WSN requires less memory than the large CNNs used for classification applications. The parameters of consequence in a WSN include the following: number of wavelet scales (J), number of wavelets per octave (Q), number of wavelet orientations (L), maximal scattering order (M), and oversampling factor (ζ). Due to its functional similarity to CNNs, a WSN can provide a benchmark for the comparison of quantization schemes. This section explores the quantization of a wavelet scattering network using a set encoding method.

For an input to a network, X, each windowed scattering propagator, $S_{J}[p]X$, for all paths p∈P, as well as the intermediate operations of the propagator, $U[\lambda_{1}]X,\;U[\lambda_{1},\lambda_{2}]X,\ldots ,\;U[p]X$, are quantized; thus, the output along a path of length T undergoes T+1 quantizations. The calculation and application of quantization levels is based on the ScatNet [45] implementation, wherein the *m*th order scattering operations, $U\left[p^{\left(m\right)}\right]X=U\left[\lambda \right]U\left[p^{\left(m-1\right)}\right]X$, and the windowing operations for the previous scattering order, $S_{J}\left[p^{\left(m-1\right)}\right]X$, are applied in one iteration of the scattering code.

The outputs, $𝑦=\left(U\left[p^{\left(m\right)}\right]X\forall p^{\left(m\right)}\in P,S_{J}\left[p^{\left(m-1\right)}\right]X\forall p^{\left(m-1\right)}\in P\right)$ from this scattering layer, or s-layer, are fed to a quantization layer, or q-layer, wherein each input, Y∈𝑦, is quantized to *K* unique levels. These quantization levels are generated using the values in *Y*, such that each windowed scattering propagator with q-layers is defined using a quantization operator Q:

(9) $$S_{J}\left[p\right]X=QS_{J}QU\left[\lambda_{m}\right]QU\left[\lambda_{m-1}\right]\ldots QU\left[\lambda_{2}\right]QU\left[\lambda_{1}\right]QX.$$

**Figure 3:**
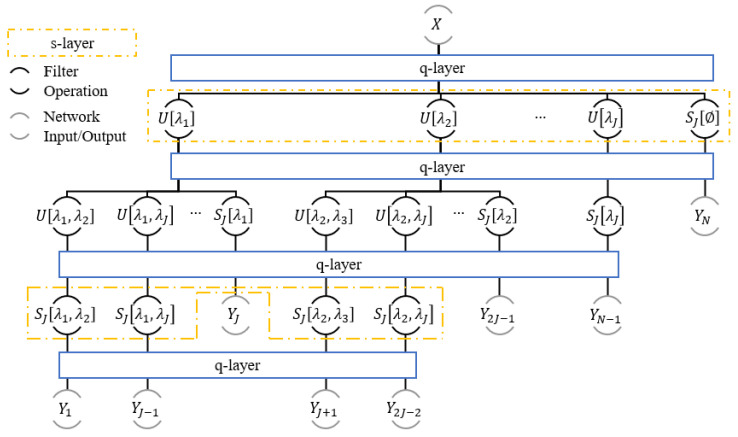
Location of the q-layers in an M=2 WSN where L=1.

Figure 3 provides a depiction of the s-layers and their corresponding q-layers for an M=2 network with L=1. First, the input X is quantized, then passed to the first s-layer (m=1). The output from the operation with $U\left[\lambda_{1}\right]$ is then quantized; the output from the operation with $U\left[\lambda_{2}\right]$ is then quantized; etc. Lastly, the output from the operation with $S_{J}\left[\phi \right]$ is quantized. This process is repeated for each scattering order of the network. Note that the quantization processes within all q-layers in a network are isolated events.

The purpose of this quantization scheme is to provide an initial comparison for quantization by limiting the number of unique values permitted. The effectiveness of such a quantization scheme is dependent upon the size of each Y∈𝑦. Suppose that |Y|=N for an s-layer. If each value in *Y* requires *b* bits for representation, then the total number of bits required to represent all V values in Y is Vb. However, the sizes of the N outputs contained in 𝑦 vary with the scales of the filters used in their computation, with the maximum number of values $\hat{V}$ in Y given by $\hat{V}\leq D_{X}$, where $D_{X}$ is the number of values in the input to the network, X. In addition, the number of outputs in 𝑦 varies with the depth of the s-layer in the network.

**Figure 4:**
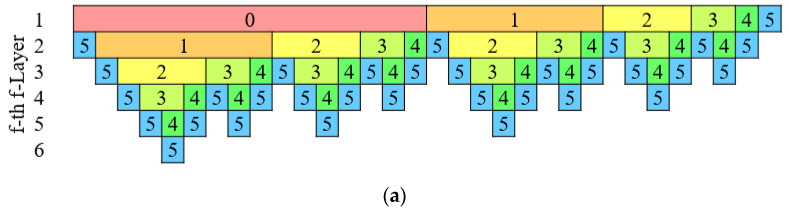

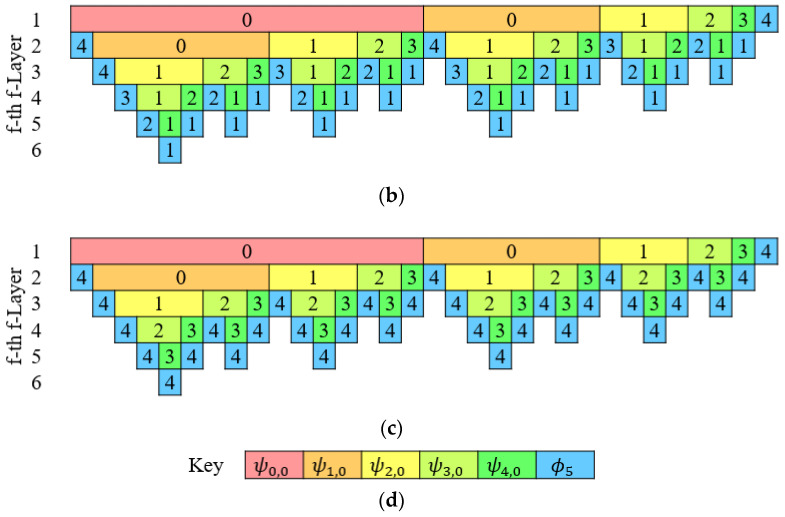
Example of a quantization scheme for a WSN with J=5 and L=1 at the input of each filter for all possible s-layers in the network: (**a**) filter scales $\psi_{\lambda }$ and $\phi_{J}$; (**b**) Log_2_ subsampling rate $d_{f}$; (**c**) cumulative log_2_ subsampling rate $r_{j}=\sum d_{f}$; (**d**) color key for filter scales.

Figure 4 provides an example for a WSN for J=5, L=1, Q=1, ζ=1, and M=5. In Figure 4a, the number of calls to each scattering wavelet filter, $\psi_{\lambda }$, with j>1, increases and then decreases with each s-layer; $\psi_{\lambda }$ with j=1 are called once only in the first and second s-layers; $\psi_{\lambda }$ with j=0 is called only once in the first s-layer. The windowing filter, $\phi_{J}$, is called during each s-layer, once for each call to a $\psi_{\lambda }$ in the previous s-layer and once in the first s-layer. The log_2_ subsampling rate of each output, $d_{f}$, provided in Figure 4b, is used to determine the size of the output from each filter, $D_{x}/2^{d}$, where $D_{x}$ is the number of elements in the input. The size of the output along any path is quantified as the sum of the log_2_ down-sampling rates or the log_2_ resolution of the output, which is shown in Figure 4c.

For a general network with Q=1, the size of the output images and the number of outputs from an s-layer are derived in the following two subsections.

### 3.1. Sizes of Filter Outputs

The input along a path of length T, $p^{\left[T\right]}$, undergoes T+1 operations; that is, the input passes through T bandpass filters associated with the operators $U[\lambda_{t}]$, $\psi_{\lambda_{1}},\psi_{\lambda_{2}},\ldots ,\psi_{\lambda_{t}},\ldots ,\psi_{\lambda_{T}}$, with scales $2^{j_{t}}\;(j_{t}\;=\;0,1,\ldots ,J-1)$ and one windowing filter, *ϕ_J_*, with scale 2^*J*^. For simplicity, the *f*th intermediate output along a scattering propagator $U\left[p^{\left(T\right)}\right]X$ is denoted as $U\left[p_{f}^{\left[T\right]}\right]X$, such that $U\left[p_{f}^{\left[T\right]}\right]X=U\left[p^{\left(f\right)}\right]X$, where *f* ≤ *T*.

Setting *Q* = 1, the resolution, $r_{\psi_{\lambda }}^{\left(f\right)}$, of a filter used in the *f*th s-layer reduces to the scale of the filter, *j_f_*, thereby reducing the log_2_ subsampling rate, *d_f_*, to [48]

(10) $$d_{f}=\left\{ \begin{array}{cc} 0, & j_{f}<r_{X}^{\left(f\right)}+\zeta \\ j_{f}-r_{X}^{\left(f\right)}-\zeta , & j_{f}\geq r_{X}^{\left(f\right)}+\zeta \end{array}\right.,$$

where $r_{X}^{\left(f\right)}$ is the resolution of the input to the *f*th filter.

Equation (4) can be reduced by examining the two cases of Equation (10). In the first case, where $j_{f}<r_{X}^{\left(f\right)}-\zeta$, both *d_f_* and $r_{X}^{\left(f\right)}$ are always 0; this allows the simplification of the case statement to *j_f_* < *ζ*. For the second case, where $j_{f}\geq r_{X}^{\left(f\right)}-\zeta$, substituting Equation (10) into Equation (4) yields

(11) $$r_{X}^{\left(f+1\right)}=j_{f}-\zeta .$$

This result is independent of $r_{X}^{\left(f\right)}$, and therefore independent of the scales of the previous filters along the path. This simplifies the case statement to *j_f_* ≥ *ζ*.

Similarly, the resolution of the output from the *f*th filter in a path is given by

(12) $$r_{X}^{\left(f+1\right)}=\left\{ \begin{array}{cc} 0, & j_{f}<\zeta \\ j_{f}-\zeta , & j_{f}\geq \zeta \end{array}\right..$$

Since $r_{X}^{\left(f+1\right)}$ depends only upon the most recent filter scale, the notation $r_{j}=r_{X}^{\left(f+1\right)}$ will denote the resolution of the output from a filter with scale 2^*j*^ for any *f*.

As described in Equation (3), the resolution of the output is the summation of the log_2_-down-sampling rate, *d_n_*, for 1 ≤ *n* ≤ *f*. The final resolution of the output, $r_{X}^{\left(T+2\right)}$, i.e., a feature map, is the summation of *d_f_* for 1 ≤ *f* ≤ *T* + 1. Therefore, for a network input, $X\in R^{D_{1}\times D_{2}}$, the number of values in the output, *Y*, from the *f*th s-layer is

(13) $$V_{j}^{\left(f\right)}=\frac{D_{1}D_{2}}{2^{2\left(r_{j}+1\right)}},$$

where the +1 is attributed to the down-sampling by the *ϕ_J_*. The size of an output is independent of the s-layer in which it is produced, again depending upon only the resolution of the output. To reduce the number of unique values in *Y*, the number of levels $K<V_{j}^{\left(f\right)}$.

The total number of values, *V*^(*f*)^, submitted from the *f*th s-layer to the following q-layer is given by

(14) $$V^{\left(f\right)}=A^{\left(f\right)}R^{\left(f\right)}+\sum_{J}V_{j}^{\left(f\right)}N_{j}^{\left(f\right)},$$

where $N_{j}^{\left(f\right)}$ is the number of outputs from each bandpass filter with a scale 2^*j*^, and *A*^(*f*)^ is the number of values in each of the *R*^(*f*)^ lowpass filter outputs.

### 3.2. Number of Filter Outputs per s-Layer

The total number of s-layers in the network is equal to *M* + 1. The number of outputs from a filter in each s-layer varies with the maximum scale, *J*, the number of orientations per scattering wavelet, *L*, and the scattering order, *m*, associated with the operations in an s-layer. In the *f*th s-layer, layer inputs undergo the operations $U\left[p^{\left(m\right)}\right]\forall p^{\left(m\right)}\in P$ and $S_{J}\left[p^{\left(m-1\right)}\right]\forall p^{\left(m-1\right)}\in P$.

If *M* = 0, then the total number of operations in the first s-layer, and the entire network, is one. However, if *M* > 0, the total number of outputs equal the number of filters. Therefore,

(15) $$N^{\left(1\right)}=\left\{ \begin{array}{cc} J\cdot L+1, & M>0\\ 0, & M=0 \end{array}\right..$$

The second s-layer accepts *N*^(1)^ − 1 outputs from the first layer $U\left[p^{\left(1\right)}\right]X\forall p^{\left(1\right)}\in P$ as inputs to the filters ($S_{J}\left[p\right]X$ constitutes a terminated path for any *p*). The windowing filter operates on all *J* · *L* inputs to the s-layer, while the bandpass filters only operate on those inputs with paths ending with a smaller scale: An input with path $p^{\left(1\right)}=\left((j_{2},\theta \in \Theta )\right)$ is operated upon by each *ψ_λ_*, where *λ* = (*j*, *θ* ∈ Θ) with *j*_2_ + *Q* ≤ *j* < *J*. Using *a_j_* = *ψ_λ_* and *L* = 1, the total number of outputs from the *ψ_λ_* filters can be stated as a function of *j* as follows,

(16) $$\begin{array}{cc} \sum_{k=0}^{J-2}\sum_{j=k+1}^{J-1}a_{k} & =(a_{1}+a_{2}+\ldots +a_{J-2}+a_{J-1})+(a_{2}+\ldots +a_{J-2}+a_{J-1})+\ldots (a_{J-2}+a_{J-1})+(a_{J-1})\\ & =a_{1}+2a_{2}+\ldots +(J-2)a_{J-2}+(J-1)a_{J-1}\\ & =\sum_{m=0}^{J-1}ka_{k}\\ & =\sum_{m=0}^{J-1}N_{k}^{\left(2\right)}a_{k} \end{array}.$$

For *L* > 1, the number of outputs from each *ψ_λ_* is $N_{j}^{\left(2\right)}=jL$. Therefore, the total number of outputs from the second s-layer is

(17) $$N^{\left(2\right)}=\left(N^{\left(1\right)}-1\right)+L\cdot \overset{J-1}{\underset{k=0}{\sum }}k.$$

If *M* > 2, then this pattern continues, with each of the *N*^(*f*−1)^ − *N*^(*f*−2)^. inputs to an s-layer. The number of outputs from any bandpass filter in the s-layer is

(18) $$N_{j}^{\left(f\right)}=\left\{\begin{array}{cc} L\cdot \overset{j-1}{\underset{k=0}{\sum }}N_{k}^{\left(f-1\right)}, & f>1\\ 1, & f=1 \end{array}\right..$$

The summation may also be expressed in terms of ${N_{j}^{\left(f\right)}|}_{L=1}$, the number outputs from a filter in the *f*th layer for *L* = 1 as 

(19) $$\begin{array}{cc} L\cdot \sum_{k=0}^{j-1}N_{k}^{\left(f-1\right)} & =L\cdot \sum_{k_{f}=0}^{j-1}\left(L\cdot \sum_{k_{f-1}=0}^{k_{f}-1}\ldots \left(L\cdot \sum_{k_{1}=0}^{k_{2}-1}N_{k_{1}}^{\left(1\right)}\right)\right)\\ & =L^{\left(f-1\right)}\cdot \sum_{k=0}^{j-1}{N_{k}^{\left(f-1\right)}|}_{L=1} \end{array}.$$

Furthermore, because *j_f_* > *j*_*f*−1_, we have $N_{j}^{\left(f\right)}=0$ for *j* < *f* − 1, which modifies Equation (20) to

(20) $$N_{j}^{\left(f\right)}=\left\{ \begin{array}{cc} L^{\left(f-1\right)}\cdot \sum_{k=f}^{j-1}N_{k}^{\left(f-1\right)}{|}_{L=1}, & j\geq f-1>0\\ 0, & j<f-1\\ 1, & f=1 \end{array}\right..$$

The total number of outputs for an s-layer is therefore [48]

(21) $$N^{\left(f\right)}=\left\{ \begin{array}{cc} N^{\left(f-1\right)}-N^{\left(f-2\right)}, & f=M+1\\ N^{\left(f-1\right)}-N^{\left(f-2\right)}+\sum_{k=f}^{j-1}N_{k}^{\left(f\right)}, & 1<f\leq M\\ 1, & f=1 \end{array}\right..$$

### 3.3. Quantization Scales

We explore several quantization scales to create *K* quantization levels. The quantization levels for *Y* ∈ 𝑦 are denoted as *v_Q_*.

#### 3.3.1. Uniform Scale

The uniform, or linear, quantization scale provides a good performance benchmark. The uniform scale was constructed by uniformly spacing values, such that the *K* levels are

(22) $$v_{Q}\in \left(\min(Y),\min(Y)+dv_{Q},\min(Y)+2dv_{Q},\ldots ,\max(Y)\right),$$

where $dv_{Q}=(\max(Y)-\min(Y)+1)/2^{K}$.

#### 3.3.2. Log Scale

Log-scale quantization provides another simple benchmark; however, to prevent values *v_Q_* ∈ *Y* that exist outside the domain of the log function, a value *dz* must be added to all values in *Y*. This results in the transformation *Y* → *Z*:*z* = log(*v* + *dz*)∀*z*∈*Z*. The log-scale is constructed as

(23) $$Z_{Q}\in \left(\min(Z),\min(Z)+dv_{Q},\min(Z)+2dv_{Q},\ldots ,\max(Z)\right),$$

(24) $$v_{Q}\in \left(e^{\min(Z_{Q})+dv_{Q}},e^{\min(Z_{Q})+2dv_{Q}},\ldots ,e^{\max(Z_{Q})}\right)$$

where $dv_{Q}=\left(\max(Y)-\min(Y)+1\right)/2^{K}$. To prevent dealing with unreasonably large numbers (which would require more memory for representation), the shifting value dz=1.

#### 3.3.3. K-Means Scale

Quantization via k-means clustering is a common method. In this paper, the k-means scaling was implemented using Lloyd’s algorithm with random initializations for *K* centroids, or quantization levels. Following the convergence of the clustering algorithm, the nearest neighbor method maps the values in *Y* to the *K* quantization levels. This method assumes convergence for the success of this quantization scale. Therefore, convergence to a global minimum is improved with successful clustering by proper selection of centroid initializations using the k-means++ algorithm [49], which uses a random number generator (RNG) for randomized seeding. The k-means++ algorithm is O(logk).

#### 3.3.4. Probability Distribution Scale

To explore the impact of the data on the quantization levels, the probability distribution function (PDF) and the output from each s-layer are used to generate quantization levels. Best-fit PDFs for the data were selected using maximum likelihood estimation (MLE). The global best-fit PDF was determined by assessing the individual fits to the s-layer outputs for each class.

The inverse Gaussian and the gamma distributions were selected to generate quantization scales. At each s-layer, the data in *Y* are fitted to one of these distributions. Quantization levels are then determined using an RNG, until *K* unique levels are found. Values were generated using the algorithms presented in [50,51] for the inverse Gaussian and the gamma scales, respectively. Lastly, each y∈Y is mapped to a level using nearest neighbor. Note that the computational time required to generate the levels for these scales is dependent on the number of unique values required, which may decline with increasing scattering order.

As with the log-scale, domain restrictions must be handled. The inverse Gaussian distribution has support on [0, ∞), therefore shifting the data such that min(*y* ∈ *Y*) ≥ 0 aids in obtaining the fitted PDF. The support of the gamma distribution, (0,∞) requires shifting the values such that min(y∈Y)>0; the value of *dz* should be as small as possible. Following the calculation and application of the quantization levels, the data are shifted by −*dz*.

#### 3.3.5. Quantile Scale

Another PDF-based quantization scale is considered that requires no RNG. The distribution of the values in *Y* are divided into *K* quantiles. The midpoint of each quantile is then used as a quantization level, *v_Q_*. This provides a more static fit of a PDF to the data; moreover, there are no support considerations required for its implementation.

## 4. Quantized Wavelet Scattering Network Results

The performances of the quantization method and scales were tested using a second-order WSN with Morlet wavelets at five scales (J=5) and eight orientations (*L* = 8) and the Gaussian windowing function. 

The Morlet wavelet is defined as

(25) $$\psi (\vec{u})=\left(e^{j\xi x}-K\right)\exp\left(-\frac{x^{2}+s^{2}y^{2}}{2\sigma_{\psi }^{2}}\right),$$

where *s* is the slant or eccentricity of the elliptical Gaussian envelope, $\sigma_{\psi }$ is the standard deviation of the elliptical Gaussian envelope, *ξ* is a parameter which permits a trade-off between the *x*- and *y*-resolutions, and *K* is a constant to ensure that the average value of $\psi (\vec{u})$ is zero.

The Gaussian windowing function is represented as

(26) $$\phi (\vec{u})=\exp\left(-\frac{x^{2}+y^{2}}{2\sigma_{\phi }^{2}}\right),$$

where $\sigma_{\phi }$ is its standard deviation.

The parameter values of the mother Morlet wavelet and the Gaussian windowing function, as defined in Equations (25) and (26), are provided in Table 2. The size of the filters at *r* = 0 was 144 × 144 pixels.

The output of the WSN is modified to form a feature vector $\vec{a}\in {\mathbb{R}}^{\sum N^{\left(f\right)}}$, such that each element is equal to $\sum_{\forall y\in Y}y$ for a unique windowed scattering propagator. These 681 features were input to a linear kernel SVM for classification. A subset of the MSTAR dataset was used for performance analysis in MATLAB. Each quantization scale was evaluated for with 2, 4, 16, and 256 quantization levels. In addition to the input and each $S_{J}[p]$ and U[p], each feature vector was quantized; the same quantization scale was implemented at each location. For the RNG-based quantization schemes, k-means, gamma, and inverse Gaussian, the effect of random number generation was evaluated by seeding the Mersenne Twister RNG (the default in MATLAB) with 10 different seeds. The effect of noise addition was assessed for four signal-to-noise ratios (SNRs) of 2, 10, 20, and 50 dB.

After assessing the implementations of the quantization schemes, the underlying performance of the WSN-SVM was compared to that of a linear kernel SVM and ResNet18 [23] for each SNR. The ResNet18 architecture was trained from scratch using stochastic gradient descent with momentum 0.9 for a maximum of 50 epochs with batch sizes of 256; the initial learn rate 1 × 10^−4^ was scaled by a factor of 0.9 every four epochs. The described training options were loosely tailored for the infinite SNR case, then used across the remaining SNR cases.

Note that compared to the WSN-SVM architecture, both the SVM and ResNet18 are more complex classifiers. Input to the SVM is the full SAR image (64^2^ features), requiring more memory in comparison to the 681 features used in the WSN-SVM. ResNet18 is a 72-layer CNN with 11.7 million learnable parameters.

### 4.1. Description of the MSTAR Dataset and Augmentations

**Figure 5:**
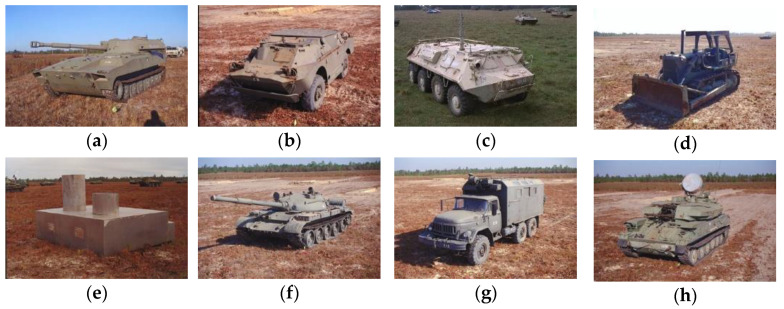
Classes in the MSTAR dataset: (**a**) 2S1; (**b**) BRDM-2; (**c**) BTR-60; (**d**) D7; (**e**) SLICY; (**f**) T62; (**g**) ZIL-131; (**h**) ZSU-23-4.

Eight of the available classes from the mixed target subset of the MSTAR database were used for classification, as shown in Figure 5. In existing works that apply CNNs to SAR data, the 15° and 17° depression angle data are used, typically separated into training and test data. While the difference between 15° and 17° may be negligible, only the 15° data were used in order to remove any possible confusion during analysis.

The number of samples from each class in the 15° subset of the MSTAR database was 274, except for the BTR-60, which was 195, totaling 2112 samples. Note that the number of samples for the BTR-60 was approximately 70% of the other classes, making this an unbalanced dataset. Each of the samples were cropped to 64 × 64 images, centered on the target, to reduce the effect of the target’s surroundings on the classification results, while retaining the entire target. The SLICY samples were uncropped, as they were only 54 × 54; to address this discrepancy, these images were symmetrically padded to the uniform size for the WSN-SVM, SVM, and ResNet18 classifiers.

**Figure 6:**
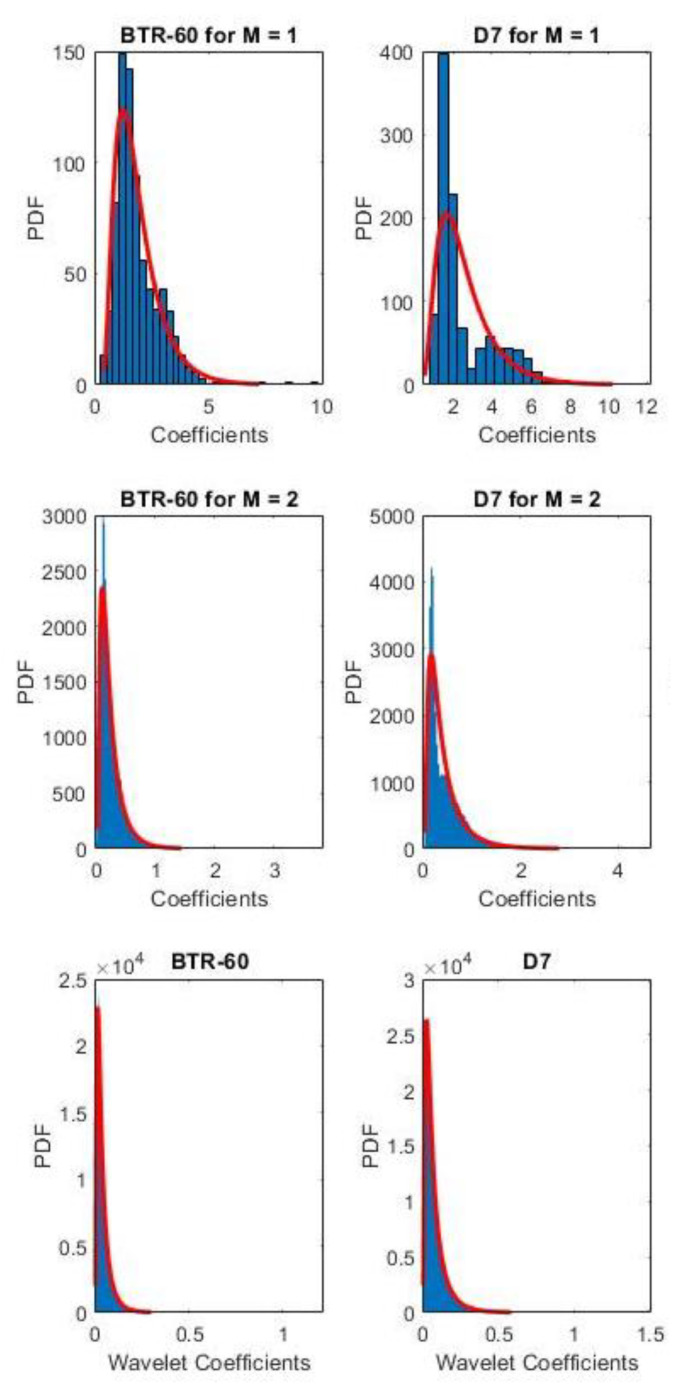
Histogram of MSTAR dataset wavelet coefficients separated by class for each s-layer in a WSN for *Q* = 1, *J* = 5, and *L* = 8: (**Top**) *M* = 1; (**Middle**) *M* = 2; (**Bottom**) fitted inverse Gaussian PDF.

Figure 6 shows the histograms and the fitting of an inverse Gaussian PDF for two example cases within the MSTAR database using the methodology described in Section 3.3.4.

To assess the effectiveness of classification in the presence of noise, white Gaussian noise, *η*, was added to the dataset prior to partitioning the samples into training and test data, such that the new noisy sample is given by $\tilde{X}=\left|\left|X\right|+\eta \right|$. Although this method of noise addition does not reflect the actual presence of noise in SAR imagery, it provides a measure to assess the robustness of the network in classifying more complex data.

### 4.2. Evaluation Metrics

For the WSN-SVM and SVM-only architectures, classification was performed using a one-vs.-all SVM with a linear kernel function. No consensus for assessing the success of a multi-class classification algorithm exists. Because the MSTAR dataset is unbalanced due to the fewer samples present for the BTR-60, the balanced accuracy was calculated rather than traditional binary calculation of accuracies. We employed the balanced accuracy, ACC, given by [52]

(27) $$ACC=\frac{1}{2}(\bar{TPR}+\bar{TNR}),$$

where $\bar{TPR}$ and $\bar{TNR}$ are the true positive and negative rates, respectively. This was expanded to a multi-class classifier implementation by calculating the balanced accuracy for each class, with the true positive and negative rates calculated as macro-averages. To accommodate the small size of the dataset, 20-fold validation was used.

## 5. Results and Discussion

### 5.1. Effects of RNG Seeding

**Figure 7:**
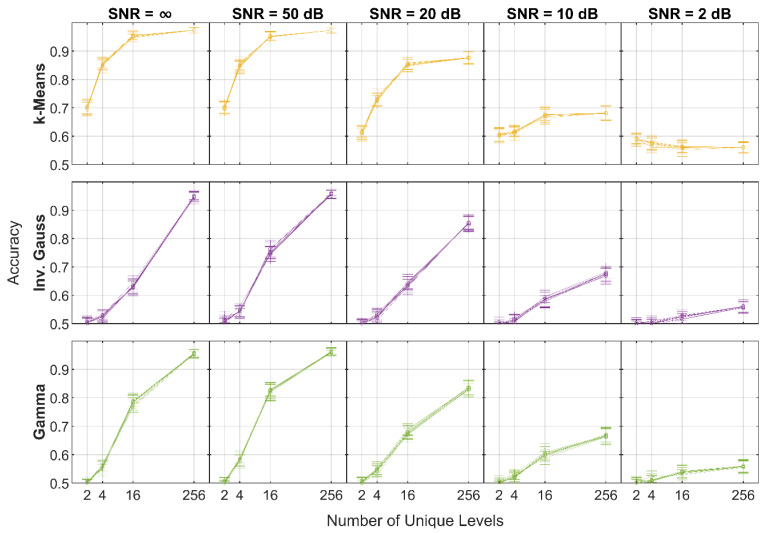
Accuracy of the networks implementing RNG-based quantization scales for 10 different initial seeds.

Figure 7 shows the results of the RNG-based quantization scales under each SNR condition for 10 different seeds. Regardless of the number of quantization levels, the variability in network performance was negligible in all cases. Of the three quantization scales, k-means outperformed the PDF-based methods for unique quantization levels 2, 4, and 16 for SNR > 10 dB; however, for SNR ≤ 10 dB, the k-means scale only outperformed the others for two and four levels.

To simplify discussion in the remainder of this paper, the performance of the RNG-based methods uses the average performance and error.

### 5.2. Noiseless and Noisy Datasets

**Figure 8:**
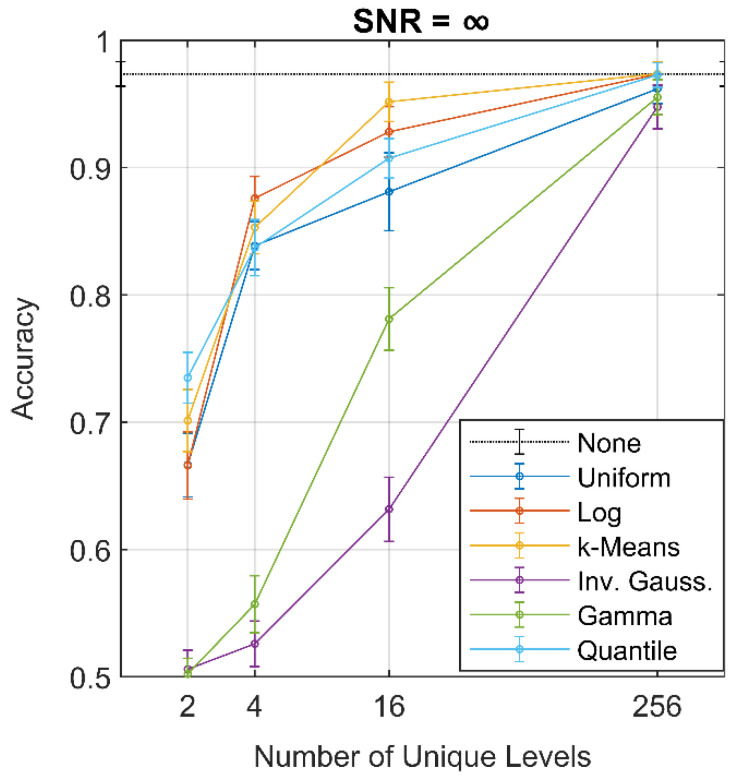
Accuracy of the quantizer scales for SNR = ∞.

The effect of quantization on the noiseless dataset for all values of *K* assessed is shown in Figure 8. As expected, a smaller *K* yielded poorer accuracy. The k-means and quantile scales performed best for two levels on average, with only the quantile scale performing significantly better than the uniform, log, and PDF-based distributions. In addition, the quantile scale had the smallest standard deviation (0.0199) for two levels, excluding the gamma and inverse Gaussian scales. However, as the number of levels increases, the quantile scale falls behind the k-means and log scales until 256 levels, where performances of these three are similar to that of the non-quantized dataset.

The steepest decrease in accuracy occurred in the gamma and inverse Gaussian scale between *K* = 256 and *K* = 16. This was likely caused by poor choice of the quantization values at the input and the first s-layer. The input to the network contained 64^2^ = 4096 values, which were quantized to *K* unique values. For *K* = 256, the maximum number of unique quantization levels is 256; if all 4096 values of the input are unique and all 256 levels exist in the input, then only 256/4096 = 0.0625 of the input is accurately represented, with the remaining 0.9375 of the input quantized. For *K* = 16, the fraction of unique quantization levels to unique values in the input reduces to 0.0039. Poor selection of initial quantization levels would, therefore, have a significant impact throughout the network. In addition, by quantizing to fewer levels, the output from the following layer likely loses the distribution shape found in the non-quantized data, such that the pre-selected PDF is no longer the best-fit within the WSN.

Due to the comparatively high accuracy of the quantile scale (nearing 0.75 even at two levels), one of the underlying causes of the poor performance of the gamma and inverse Gaussian quantization scales was likely the RNG employed to generate the quantization levels. As there is no guarantee that the output of each q-layer retains its shape, as shown in Figure 6, the estimated PDF is not necessarily a best-fit function. While an RNG is also core to the k-means scale, the initial centroids were heuristically selected to improve performance per the k-means++ algorithm, whereas the other RNG-based scales presuppose the underlying PDF.

**Figure 9:**
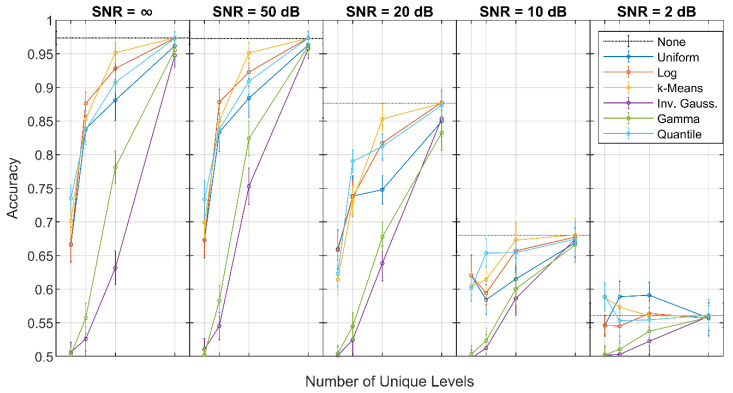
Accuracy of the quantizer scales for infinite, 50, 20, 10, and 2 dB SNR.

As the SNR decreased, the quantized WSN-SVMs generally experienced the same decrease in accuracy as the non-quantized WSN-SVMs at *K* = 256, as shown in Figure 9. The results indicate that only 256 quantization levels are required for a quantized WSN-SVM to achieve similar performance to the non-quantized network, regardless of SNR, due in part to the decreasing size of the outputs of each filter: the majority of filters in the network produce outputs containing 256 or fewer values. (This can be quickly verified from the log_2_ subsampling rates chart in Figure 4b.) The number of unique values is only reduced at the scattering propagator U[p] for p=Ø, ((0,θ)), ((0,θ),(1,θ)), and ((1,θ)) for all θ∈Θ. Therefore, the performance of each quantized WSN-SVM is likely more affected by appropriate selection at the majority of quantization locations. At *K* = 256, the average accuracy of the k-means, log, and quantile scales typically coincided with the non-quantized network, although the error increased with decreasing SNR.

For *K* < 256, the quantile scale appeared to better retain its performance relative to that of the non-quantized WSN-SVM at each SNR; that is, the accuracy of the quantile scale at these levels decreased less with SNR than other scales, although performance typically remained comparable with the k-means and log scales. For the 2 dB SNR case, the quantile, k-means, and uniform scales outperformed the non-quantized data, which might indicate some inherent trend in the data, despite the noise addition.

### 5.3. Comparison with the SVM and ResNet18

The results of the non-quantized WSN-SVM, SVM, and ResNet18 for all five SNR cases is provided in Table 3. The WSN-SVM architecture performed comparably to the standard SVM, with the WSN-SVM constituting a less complex training process with only 681 features input to its SVM compared to the 4096 features of the standard SVM. Apart from outperforming traditional SVM, Table 3 shows that WSN-SVM substantially outperformed ResNet18 for the infinite, 50, and 20 dB SNR cases. Since the considered SNR regime (around 20 dB) is operationally significant in many applications, WSN-SVM can thus be a potentially powerful alternative to both CNNs and traditional ML approaches such as SVM in such scenarios. Furthermore, even though WSNs do not perform as well as CNNs in the low-SNR regime, the computational complexity to train WSNs is substantially less as well.

The poorer performance of ResNet18 in the mid- to higher-SNR regimes could be attributed to the implemented training method and associated parameters. However, for the 10 and 2 dB SNR cases, the accuracy of ResNet18 dropped by approximately 0.04 and 0.06, respectively, from that of the infinite SNR, whereas the WSN-SVM dropped by approximately 0.29 and 0.43. The robustness of ResNet18 to decreasing SNR may be attributed to both the adaptability of the network, as well as its depth and the number of filters per layer.

Note that though it does not contain learnable parameters, the wavelet parameters of the WSN presented in Table 2 may also be adjusted to improve performance.

## 6. Conclusions

Due to its structural similarity with CNNs, the WSN-based quantization studies performed in this study may provide a good benchmark for future work in the quantization of CNN-based neural networks. We explored the classification accuracy of quantized WSNs with a multi-class SVM. We overcame the limited performance of RNG-based PDF quantization schemes by incorporating data-driven methods for selecting the quantization levels as indicated by the performance of k-means and quantile schemes. The deleterious effects of compounding of error resulting from poorly selected quantization levels and the limitations of RNG-based PDF quantization scaling were overcome by devising RNG-based k-means scaling and statically generated levels of the PDF-based quantile scales. The performance of various quantization methods was quantitatively studied for different levels of noise, which can provide guidance for the design of quantized CNNs under practical operating scenarios. Based on the comparison of the WSN-SVM with ResNet18, future work should investigate the application of the quantization scheme in ResNet18 and similar CNN architectures.

## Figures and Tables

**Table 1:** System parameters for WSN.

*M*	*J*	*L*	Number of Paths
0	-	-	1
1	3	1	4
1	3	2	7
1	3	2	25
1	5	1	6
1	5	2	11
1	5	8	41
2	3	1	7
2	3	2	19
2	5	1	16
2	5	2	51

**Table 2:** Parameter values of mother Morlet wavelet and Gaussian windowing function.

Parameter	Value
*σ_ϕ_*	0.8
*σ_ψ_*	0.8
*S*	0.5
*ξ*	2.356

**Table 3:** Performance accuracies of non-quantized WSNs, SVMs, and ResNET18.

SNR (dB)	WSN-SVM	SVM	ResNet18
∞	0.974 ± 0.0071	0.973 ± 0.011	0.818 ± 0.0098
50	0.973 ± 0.0053	0.972 ± 0.011	0.819 ± 0.011
20	0.871 ± 0.013	0.835 ± 0.021	0.799 ± 0.016
10	0.681 ± 0.020	0.659 ± 0.024	0.774 ± 0.027
2	0.557 ± 0.015	0.571 ± 0.023	0.754 ± 0.024

## Data Availability

Not applicable.
